# 721 The Rising Use of Fire as a Weapon

**DOI:** 10.1093/jbcr/irad045.196

**Published:** 2023-05-15

**Authors:** Jason Woods, Jeffrey Carter, Kathe Conlon, Carl Flores, Randy Kearns

**Affiliations:** International Association of Firefighters, Washington, District of Columbia; University Medical Center New Orleans & LSUHSC, River Ridge, Louisiana; The Burn Center at Cooperman Barnabas, West Orange, New Jersey; University Medical Center - New Orleans, Ponchatoula, Louisiana; University of New Orleans, New Orleans, Louisiana; International Association of Firefighters, Washington, District of Columbia; University Medical Center New Orleans & LSUHSC, River Ridge, Louisiana; The Burn Center at Cooperman Barnabas, West Orange, New Jersey; University Medical Center - New Orleans, Ponchatoula, Louisiana; University of New Orleans, New Orleans, Louisiana; International Association of Firefighters, Washington, District of Columbia; University Medical Center New Orleans & LSUHSC, River Ridge, Louisiana; The Burn Center at Cooperman Barnabas, West Orange, New Jersey; University Medical Center - New Orleans, Ponchatoula, Louisiana; University of New Orleans, New Orleans, Louisiana; International Association of Firefighters, Washington, District of Columbia; University Medical Center New Orleans & LSUHSC, River Ridge, Louisiana; The Burn Center at Cooperman Barnabas, West Orange, New Jersey; University Medical Center - New Orleans, Ponchatoula, Louisiana; University of New Orleans, New Orleans, Louisiana; International Association of Firefighters, Washington, District of Columbia; University Medical Center New Orleans & LSUHSC, River Ridge, Louisiana; The Burn Center at Cooperman Barnabas, West Orange, New Jersey; University Medical Center - New Orleans, Ponchatoula, Louisiana; University of New Orleans, New Orleans, Louisiana

## Abstract

**Introduction:**

Disaster planning and preparedness for a Burn Mass Causality Incident (BMCI) must consider both worst-case scenarios as well as most likely events that could strain a burn center (BC) resources. This study evaluates the growing threat of utilizing fire as a weapon for terrorist attacks and criminal acts in the United States.

**Methods:**

Statistics regarding the use of fire as a weapon over a 2-year period were reviewed and summarized along with a review of significant events over 30 years. In addition, data regarding the type of weapon used in these attacks, intended target, and motives were collected and compared.

**Results:**

According to the Cyber Security and Infrastructure Security Agency (CSISA), incidents related to arson or fire as a weapon in the US increased by 56.9% in cities with populations over 1 million people between 2019 and 2020 and 11.3% in towns with populations under 10,000. In addition, improvised Incendiary Device (IID) incidents increased by 210% at government facilities, 113% at commercial facilities, and 114% at critical infrastructure facilities during the same time frame.

**Conclusions:**

The US continues to face complex and evolving threats from extremists, criminals, and terrorists. Methods have included attacks utilizing arson, IIDs, deliberate forest fires, and more. Assailants have used fire as a weapon to target public gatherings, individuals, and government property and as a component of complex coordinated attacks. These attacks have resulted in both large numbers of fatalities and burn injuries. The materials required to create a weapon utilizing fire are cheap, readily available, and require no formal training.

**Applicability of Research to Practice:**

Criminal or terrorism-related incidents involving the use of fire as a weapon can result in mass causality events with large numbers of burn patients. These incidents can potentially overwhelm both first responders and local BC quickly. Therefore, BCs must have an updated disaster plan that has been evaluated and communicated with local fire and EMS, neighboring hospitals, and regional BCs.

